# Point-of-care testing for pre-hospital stratification after out-of-hospital cardiac arrest: the RAPID-MIRACLE study

**DOI:** 10.1093/ehjacc/zuag036

**Published:** 2026-03-30

**Authors:** Muhamad Abd Razak, Prajith Jeyaprakash, Michael McGarvey, John Hodsoll, Evan Ansell, Roman Roy, Krishnaraj Rathod, Shayna Chotai, Fiyyaz Ahmed-Jushuf, Oliver Rees, Vasileios Panoulas, Miles Dalby, Sundeep Kalra, Tiffany Patterson, Sami Firoozi, Iqbal Malik, Paul Rees, Rafal Dworakowski, Ajay M Shah, Daniel Stahl, Philip MacCarthy, Jon Byrne, Adam Mellett-Smith, Rachael Fothergill, Nilesh Pareek

**Affiliations:** Department of Cardiology, King’s College Hospital NHS Foundation Trust, Denmark Hill, London SE5 9RS, UK; BHF Centre of Excellence, School of Cardiovascular Medicine and Metabolic Sciences, James Black Centre, 125 Coldharbour Lane, King’s College London SE5 9NU, UK; Department of Cardiology, King’s College Hospital NHS Foundation Trust, Denmark Hill, London SE5 9RS, UK; Department of Cardiology, Nepean Hospital, Kingswood, New South Wales, Australia; Department of Cardiology, King’s College Hospital NHS Foundation Trust, Denmark Hill, London SE5 9RS, UK; BHF Centre of Excellence, School of Cardiovascular Medicine and Metabolic Sciences, James Black Centre, 125 Coldharbour Lane, King’s College London SE5 9NU, UK; Department of Biostatistics and Health Informatics, Institute of Psychiatry, Psychology and Neuroscience, King’s College London, London, UK; Department of Cardiology, King’s College Hospital NHS Foundation Trust, Denmark Hill, London SE5 9RS, UK; BHF Centre of Excellence, School of Cardiovascular Medicine and Metabolic Sciences, James Black Centre, 125 Coldharbour Lane, King’s College London SE5 9NU, UK; Department of Cardiology, King’s College Hospital NHS Foundation Trust, Denmark Hill, London SE5 9RS, UK; Barts Heart Centre, Barts Health NHS Trust, London, UK; Department of Cardiology, Imperial College Healthcare NHS Foundation Trust, London, UK; Department of Cardiology, Imperial College Healthcare NHS Foundation Trust, London, UK; Department of Cardiology, St.George’s University Hospitals NHS Foundation Trust, London, UK; Department of Cardiology, Royal Brompton and Harefield Hospitals, Guy’s and St. Thomas’ NHS Foundation Trust, Harefield Hospital, UK; Department of Cardiology, Royal Brompton and Harefield Hospitals, Guy’s and St. Thomas’ NHS Foundation Trust, Harefield Hospital, UK; Department of Cardiology, Royal Free Hospital NHS Foundation Trust, London, UK; Department of Cardiology, Guy’s and St.Thomas’ NHS Foundation Trust, London, UK; Department of Cardiology, St.George’s University Hospitals NHS Foundation Trust, London, UK; Department of Cardiology, Imperial College Healthcare NHS Foundation Trust, London, UK; Barts Heart Centre, Barts Health NHS Trust, London, UK; Center for Resuscitation Medicine, University of Minnesota, Minneapolis, USA; Department of Cardiology, King’s College Hospital NHS Foundation Trust, Denmark Hill, London SE5 9RS, UK; BHF Centre of Excellence, School of Cardiovascular Medicine and Metabolic Sciences, James Black Centre, 125 Coldharbour Lane, King’s College London SE5 9NU, UK; Department of Cardiology, King’s College Hospital NHS Foundation Trust, Denmark Hill, London SE5 9RS, UK; BHF Centre of Excellence, School of Cardiovascular Medicine and Metabolic Sciences, James Black Centre, 125 Coldharbour Lane, King’s College London SE5 9NU, UK; Department of Biostatistics and Health Informatics, Institute of Psychiatry, Psychology and Neuroscience, King’s College London, London, UK; Department of Cardiology, King’s College Hospital NHS Foundation Trust, Denmark Hill, London SE5 9RS, UK; BHF Centre of Excellence, School of Cardiovascular Medicine and Metabolic Sciences, James Black Centre, 125 Coldharbour Lane, King’s College London SE5 9NU, UK; Department of Cardiology, King’s College Hospital NHS Foundation Trust, Denmark Hill, London SE5 9RS, UK; BHF Centre of Excellence, School of Cardiovascular Medicine and Metabolic Sciences, James Black Centre, 125 Coldharbour Lane, King’s College London SE5 9NU, UK; Clinical Audit and Research Unit, London Ambulance Service NHS Trust, London, UK; Clinical Audit and Research Unit, London Ambulance Service NHS Trust, London, UK; Department of Cardiology, King’s College Hospital NHS Foundation Trust, Denmark Hill, London SE5 9RS, UK; BHF Centre of Excellence, School of Cardiovascular Medicine and Metabolic Sciences, James Black Centre, 125 Coldharbour Lane, King’s College London SE5 9NU, UK

**Keywords:** Out-of-hospital cardiac arrest, MIRACLE_2_ score, ROSC-MIRACLE_2_, Risk stratification, Neurological outcome, Point-of-care testing, Return of spontaneous circulation

## Abstract

**Aims:**

Out-of-hospital cardiac arrest (OHCA) has high mortality, and outcomes remain heterogeneous despite guidelines recommending universal conveyance to cardiac arrest centres. Early pre-hospital risk stratification may identify patients most likely to benefit. The pre-hospital utility of MIRACLE_2_ is unknown, so we evaluated the feasibility of rapid point-of-care testing to enable calculation of the MIRACLE_2_ score after return of spontaneous circulation (ROSC).

**Methods and results:**

RAPID-MIRACLE was a prospective, multi-centre observational study conducted across London with the London Ambulance Service. Adult patients with suspected cardiac aetiology OHCA achieving sustained ROSC were enrolled. Pre-hospital point-of-care venous blood-gas sampling was performed with results blinded to receiving hospitals. We evaluated ROSC-MIRACLE_2_ incorporating post-ROSC pH, compared with a modified MIRACLE_2_ excluding pH (pre-MIRACLE_2_) and standard MIRACLE_2_ calculated on hospital admission. The primary outcome was poor neurological outcome at 30 days, defined as cerebral performance category (CPC) 3–5. Among 292 patients, 48% had poor neurological outcome. ROSC-MIRACLE_2_ demonstrated excellent discrimination [area under the receiver operating characteristic curve (AUC) 0.89 (95% CI 0.85–0.92)], comparable to pre-MIRACLE_2_ [AUC 0.88 (95% CI 0.84–0.92)], and admission MIRACLE_2_ [AUC 0.89 (95% CI 0.85–0.92)]. For ROSC-MIRACLE_2_, a threshold 0–2, the negative predictive value for good outcome was 0.89 (0.82–0.94). At a threshold ≥5, the positive predictive value was 0.88 (0.82–0.94). In a multi-variable regression model, post-ROSC pH was independently associated with poor neurological outcome and less than 3% of patients with a ROSC pH <7.00 had good neurological outcome.

**Conclusion:**

In this study, pre-hospital application of ROSC-MIRACLE_2_ enables early neurological risk stratification following resuscitated OHCA. Point-of-care pH improves prognostic precision, but is constrained by feasibility, whilst the simplified pre-MIRACLE_2_ score is more practical with comparable performance. Integration into OHCA care pathways may improve patient stratification and resource utilization but requires further study.

## Graphical Abstract

**Graphical Abstract:**
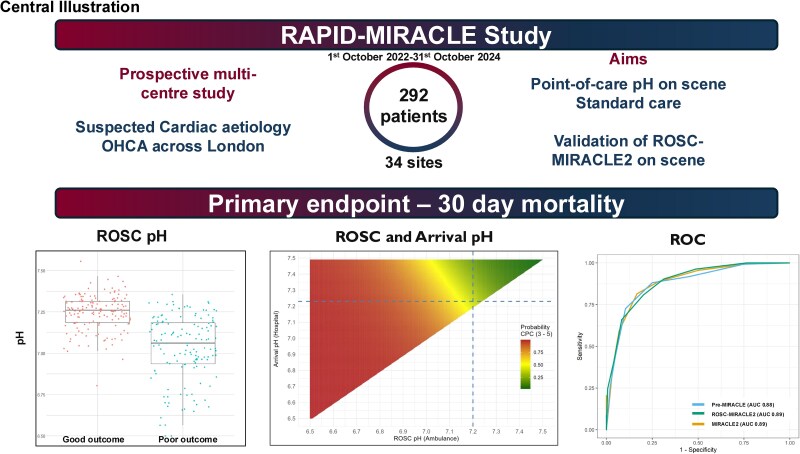

## Introduction

Out-of-hospital cardiac arrest (OHCA) is a life-threatening emergency affecting over 3 million people worldwide each year.^1,2^ Two-thirds of patients have an underlying cardiac cause, with one third of all patients presenting with an initial shockable rhythm.^1^ Despite advances in acute care, prognosis following OHCA remains poor, with fewer than 10% of patients from the community surviving to hospital discharge.^1^ Irreversible neurological injury, sustained during cardiac arrest or immediately after return of spontaneous circulation (ROSC), is the most common mode of in-hospital death.^3^ Improving outcomes for these patients have been identified as a key priority by international societies.^4–6^

Current international guidelines recommend that, whenever possible, patients with OHCA and a suspected underlying cardiac cause should be transferred to a specialist cardiac arrest centre (CAC).^7^ CACs provide 24/7 access to advanced cardiovascular assessment and specialist critical care that observational studies have suggested may improve outcomes.^8^ However, when expedited CAC transfer strategies were tested in a randomized controlled trial of resuscitated OHCA patients without ST-segment elevation on 12 lead electrocardiogram (ECG) following ROSC (the ARREST trial), no improvement in clinical outcomes was observed.^9^ These findings highlight limitations of current pre-hospital triage which suggest that undifferentiated CAC conveyance may not be beneficial. A more nuanced approach to pre-hospital stratification may enable a more effective and targeted use of CAC resources to improve outcomes.^10,11^

The MIRACLE_2_ score is a practical risk tool that was developed to predict poor neurological outcome at 6 months, in comatose patients following ROSC after OHCA. It incorporates seven variables, including blood pH, that are applied on admission to a CAC, and predicts adverse neurological outcomes with high accuracy [area under the receiver operating characteristic curve (AUC) of 0.84–0.91].^12^ When combined with an ECG and assessment of post-ROSC cardiogenic shock (CS) status [according to the Society for Cardiovascular Angiography and Interventions (SCAI) classification of CS], it appears possible to identify a cohort most likely to benefit from direct admission to a CAC and early coronary angiography.^10,13^ Whilst the MIRACLE_2_ score has been extensively validated in patients admitted to hospital, its performance in the pre-hospital setting is unknown.^14,15^ Calculation of the MIRACLE_2_ score in the community, immediately post-ROSC, requires pre-hospital blood-gas analysis (providing the pH) through rapid point-of-care testing, but the feasibility of such an approach is not known.

Accordingly, the RAPID-MIRACLE study was designed to evaluate the feasibility of rapid point-of-care testing to enable pre-hospital calculation of the MIRACLE_2_ score (henceforth referred to as the ROSC-MIRACLE_2_ score), and to prospectively validate its performance in the pre-hospital setting. Secondly, we validate the performance of a modified form of the MIRACLE_2_ score without the pH (the pre-MIRACLE_2_ score) and compare both against the MIRACLE_2_ on admission to a centre.

## Methods

### Study setting

RAPID-MIRACLE (NCT05185063) is a prospective, multi-centre observational cohort study conducted in Greater London, UK, in conjunction with the London Ambulance Service (LAS) NHS Trust. LAS deploy advanced paramedic practitioners in critical care (APP-CC) with specialist training in advanced resuscitation, airway management and point-of-care diagnostics, in addition to the standard emergency medical service (EMS) OHCA response (full description in Supplementary Material). London operates a regionalized system of care for OHCA, whereby patients achieving ROSC are conveyed to designated heart attack centres (HAC) or the nearest emergency department (ED) in accordance with national guidance. Patients with evidence of ST-segment elevation myocardial infarction (STEMI) are conveyed directly to HACs and all others are transferred initially to the local ED.^16–18^ A total of 34 hospital sites, 7 designated as HACs and 27 non-HAC, participated in the study, covering a catchment population of ∼9 million people. This included all acute hospitals capable of receiving OHCA patients within Greater London. Further details on participating centres are available in Supplementary material online, *Table S1*.

### Study population

We enrolled consecutive adult patients aged 18 years or older who sustained a non-traumatic OHCA of suspected cardiac aetiology and achieved sustained ROSC in the pre-hospital setting which was pre-defined as at least 20 min of continuous circulation following resuscitation.

Patients were excluded if there was a presumed non-cardiac cause of arrest, including trauma, drowning, suicide or drug overdose, documented or suspected pre-arrest neurological disability corresponding to cerebral performance category (CPC) 3 or 4, or a known do not attempt cardiopulmonary resuscitation order. Patients with clinically suspected or confirmed intra-cerebral bleeding, known disease limiting survival to 6 months and patients who were known or suspected to be pregnant were also excluded.

APP-CC clinicians were responsible for patient screening, enrolment, and documentation of pre-hospital study data. Post-resuscitation management, destination of hospital selection, and in-hospital care followed established clinical pathways and were not influenced by study participation. Point-of-care results were not included in the handover to the receiving centre and so were not available to admitting clinicians at the centre. The ROSC-MIRACLE_2_ and Pre-MIRACLE_2_ scores were not calculated at the point of recruitment and no aspect of subsequent clinical management was altered by study participation, including formal neuroprognostication, which was performed in accordance with current guidelines.^19^

### Data collection

Eligible patients underwent pre-hospital venous blood-gas sampling performed by the APP-CC using a point-of-care analyser. Two multi-analyte point-of-care blood-gas platforms were used during the study, the i-STAT 1, with CG4 + assay (Abbott Vascular, Illinois, USA) and the Siemens epoc NXS Host Blood Analysis System (Siemens Healthcare, Erlangen, Germany). Both devices are FDA approved and CE-marked with availability for use in routine clinical practice, requiring 2 mL of capillary, arterial or venous blood. Both systems provide pH, lactate, oxygen saturations, and basic electrolyte data with high correlation with established blood analysis systems.^20–22^ Venous blood sampling was performed using one of the platforms after sustained ROSC had been achieved and prior to hospital conveyance for study purposes only. Up to two attempts at venepuncture and analysis were carried out, with study recruitment abandoned if unsuccessful.

Data were recorded in a dedicated database using the research electronic data capture tool using Utstein definitions.^23,24^ Pre-hospital resuscitation variables, and variables required to calculate the MIRACLE_2_ score were recorded contemporaneously by LAS personnel on scene and collected in the database by research paramedics. Zero-flow time was calculated as the time from collapse or recognition of cardiac arrest to commencement of cardiopulmonary resuscitation (CPR) (by bystanders or EMS). Low-flow time was calculated as the time from commencement of CPR to ROSC, in line with Utstein guidelines.^23^ In-hospital data included early arterial blood-gases, standard biochemical and haematological measurements, and baseline cardiovascular investigations comprising ECG, emergency echocardiography and coronary angiography (where indicated).

### Outcomes

The primary outcome was poor neurological outcome at 30 days defined as CPC 3–5, in accordance with Utstein recommendations.^25^ CPC 1 denotes normal neurological function, CPC 2 denotes moderate disability with independent living, CPC 3 denotes severe disability, CPC 4 denotes persistent disorder of consciousness and CPC 5 death. Neurological outcome was assessed at follow-up using medical records and routine clinical review. Mortality outcomes were recorded for all patients. Feasibility was assessed retrospectively as the proportion of patients in whom the risk score could be calculated from the available finalized dataset. Patients were considered non-feasible if any required variable was missing, including clinical data, documented arrest characteristics, or if point-of-care analysis was not possible, including those patients excluded from the final study analysis. For reporting purposes, feasibility was defined as moderate when the score could be calculated in 50–79% of patients and high when complete data were available in at least 80% of patients.

### Ethics, governance, and consent

The study was conducted in accordance with the Declaration of Helsinki and received approval from the UK Research Ethics Committee, Confidential Advisory Group and Health Research Authority (22/EE/0001). Given the emergency nature of OHCA, the need for prospective informed consent was waived, with full details of participant recruitment and ethical approvals shown in the Supplementary Material. The study was reported in accordance with the STROBE guidelines (Supplementary material online, *Table S2*).

### Statistical analysis

Continuous variables are reported as mean ± standard deviation or median (inter-quartile range), and categorical variables as figures and percentages. Complete-case analysis was performed since <5% of data were missing. Pre-hospital and arrival pH values, along with other continuous predictors, were summarized and stratified by neurological outcome at 30 days.

Logistic regression models were fitted to assess associations between candidate predictors and poor neurological outcome, with coefficients reported for the MIRACLE_2_ score (calculated on hospital admission), ROSC-MIRACLE_2_ (calculated retrospectively based on recorded post-ROSC values), and the pre-MIRACLE2 score (with pH excluded). Model performance was evaluated using AUC, Brier score, *R*^2^, and optimism-corrected metrics from 1000 bootstrap iterations. Sensitivity, specificity, and positive and negative predictive values were calculated at clinically relevant score thresholds, as pre-defined in the original derivation and validation studies.^12^ The relationship between point-of-care pre-hospital and admission pH was explored using generalized additive models, allowing non-linear effects stratified by 30 day outcome and described as predicted curves. The optimal thresholds for both immediate post-ROSC and admission pH were identified using a plot vs. criterion analysis, plotting a two-graph receiver operating characteristic curve of the sensitivity and specificity of pH as a predictor of the primary endpoint. Finally, we performed multi-variable logistic regression to identify independent predictors of poor neurological outcome. All analyses were conducted using R version 3.5.3.

## Results

Between 1 October 2022 and 31 October 2024, a total of 1792 OHCA incidents attended by APP-CCs were assessed for eligibility. The study flowchart is illustrated in Supplementary material online, *Figure S1*. After applying exclusion criteria—including absence of ROSC, non-cardiac aetiology, age <18 years, or other clinical contraindications—521 patients underwent attempted point-of-care sampling, of which 188 (36.1%) were excluded due to device error, inability to gain venous blood sample, or failed analysis for other reasons. 41 patients declined consent or had opted out of the use of their data for research purposes prior to study enrolment. In total, 292 patients achieving ROSC after OHCA and attended by APP-CCs were recruited [median age 61 years (IQR 51–69), 82% male], of whom 80% presented with an initial shockable rhythm (*Table 1*). 246 (84%) patients had a witnessed OHCA, 227 (78%) received bystander CPR and 65 (22%) had defibrillation delivered by a public access defibrillator. The median zero-flow time was 2 min (IQR 0–4) and low-flow time was 20 min (IQR 12–32). In total, 227/292 (77.7%) of patients were conveyed directly to a HAC. On arrival at hospital, 136 (47%) had STEMI, 227 (78%) had SCAI B-E CS. 173 (59%) of patients underwent immediate (<2 h) CAG (Supplementary material online, *Tables S8*–S10). Among those recruited, 153 patients (52%) had good neurological outcome at 30 days (CPC 1–2), whereas 139 patients (48%) experienced poor outcome (CPC 3–5). Baseline demographics and clinical characteristics differed significantly between those with good and poor outcome (*Table 1*).

**Table 1:** Descriptive statistics for key demographic and clinical variables stratified by outcome

Variables	Overall (*n* = 292)^a^	Good outcome (CPC 1–2) *n* = 153^a^	Poor outcome (CPC 3–5) *n* = 139^a^	*P*-value^b^
Baseline characteristics				
Age (years)—Median (IQR)	61 (51, 69)	59 (51, 67)	63 (54, 72)	**0**.**026**
Male—no./total no. (%)	238/292 (82%)	136/153 (89%)	102/139 (73%)	**<0**.**001**
White ethnicity	193/292 (66%)	109/153 (71%)	84/139 (60%)	0.2
Asian or British Asian Ethnicity	73/292 (25%)	30/153 (20%)	43/139 (31%)	
Black, Black British, Caribbean, or African Ethnicity	11/292 (4%)	7 (2%)	4 (1%)	
Mixed or other	15/292 (5%)	7 (2%)	8 (3%)	
DM—no./total no. (%)	68/292 (23%)	28/153 (18%)	40/139 (29%)	**0**.**034**
Smoking—no./total no. (%)	58/292 (20%)	28/153 (18%)	30/139 (22%)	0.5
Hypertension—no./total no. (%)	111/292 (38%)	56/153 (37%)	55 (40%)	0.6
Previous CABG—no./total no. (%)	9/292 (3.1%)	4/153 (2.6%)	5/139 (3.6%)	0.7
Previous PCI—no./total no. (%)	38/292 (13%)	22/153 (14%)	16/139 (12%)	0.5
Known cardiomyopathy—no./total no. (%)	33/292 (11%)	10/153 (6.5%)	23/139 (17%)	**0**.**007**
Arrest characteristics				
Residence—no./total no. (%)	152/291	65/152 (42%)	87/139 (63%)	**0**.**003**
Witnessed—no./total no. (%)	246/289 (84%)	137/151 (90%)	109/138 (78%)	**0**.**011**
Bystander CPR—no./total no. (%)	227/291 (78%)	123/152 (80%)	104/139 (75%)	0.3
Public access defibrillation—no./total no. (%)	65/291 (22%)	49/152 (32%)	16/139 (12%)	**<0**.**001**
Zero-flow time (min), median (IQR)	2 (0, 4)	1 (0, 3)	2 (0, 5)	**0**.**034**
Low-flow time (min), median (IQR)	20 (12, 32)	15 (11, 20)	29 (17, 42)	**<0**.**001**
Initial shockable rhythm—no./total no. (%)	233/291 (80%)	144/152 (94%)	89/139 (64%)	**<0**.**001**
Reactive pupils—no./total no. (%)	201/291 (69%)	135/153 (88%)	66 (47%)	**<0**.**001**
Changing rhythms—no./total no. (%)	137/289 (47%)	43/151 (28%)	94/138 (68%)	**<0**.**001**
Epinephrine—no./total no. (%)	160/292 (55%)	38/153 (25%)	122/139 (88%)	**<0**.**001**
Arrest to centre time (min), median (IQR)	82 (68, 105)	73 (61, 85)	104 (82, 116)	**<0**.**001**
pH at POC testing, median (IQR)	7.19 (7.04, 7.27)	7.26 (7.18, 7.31)	7.06 (6.93, 7.19)	**<0**.**001**
Lactate at POC testing (mmol/L), median (IQR)	7.7 (5.8, 9.4)	6.4 (4.7, 8.6)	8.4 (7.1, 10.8)	**<0**.**001**
Hospital admission characteristics				
Admission pH—median (IQR)	7.24 (7.12, 7.32)	7.29 (7.23, 7.35)	7.13 (7.04, 7.25)	**<0**.**001**
pH change (admission—POC)—median (IQR)	0.04 (−0.01, 0.12)	0.03 (−0.02, 0.10)	0.06 (−0.01, 0.17)	**0**.**005**
Admission lactate (mmol/L)—median (IQR)	4.5 (2.8, 7.4)	3.5 (2.2, 5.5)	6.5 (3.9, 10.0)	**<0**.**001**
SCAI shock grade (B-E) on arrival	227/292 (78%)	99/153 (65%)	128/139 (92%)	**<0**.**001**
Serum creatinine (µmol/L)—median (IQR)	100 (79, 124)	92 (76, 110)	112 (87, 143)	**<0**.**001**
STEMI/LBBB—no./total no. (%)	136/287 (47%)	84/152 (54%)	54/135 (40%)	**0**.**018**
Immediate CAG—no./total no. (%)	173/290 (59%)	105/152 (69%)	68/138 (49%)	**<0**.**001**
PCI—no./total no. (%)	149/283 (51%)	96/149 (63%)	53/134 (38%)	**<0**.**001**
Door-to-balloon time (min), median (IQR)	63 (41, 102)	59 (37, 110)	69 (45, 90)	0.2
Multi-vessel disease no./total no. (%)	106/217 (49%)	63/136 (46%)	43/81 (53%)	0.10
MCS use no./total no. (%)	22/292 (7.5%)	8/153 (5.2%)	14/139 (10%)	0.12

Bold *P*-values indicate statistical significance (*P* < 0.05). IQR, inter-quartile range; DM, diabetes mellitus; CABG, coronary artery bypass graft; PCI, percutaneous coronary intervention; CPR, cardiopulmonary resuscitation; ECG, electrocardiogram; STEMI, ST-segment elevation myocardial infarction; LBBB, left bundle branch block; RBBB, right bundle branch block; SCAI, Society of Cardiovascular Angiography and Interventions; Immediate CAG, coronary angiogram < 2 h; CPC, cerebral performance category; POC, point-of-care testing with ambulance; Multi-vessel disease, coronary disease involving 2 or more major arterial territories; MCS, mechanical circulatory support including IABP, Impella and/or ECMO.

^a^Median (Q1, Q3); n/N (%).

^b^Wilcoxon rank sum test; Pearson’s χ^2^ test; Fisher’s exact test.

### Association between ROSC pH and poor neurological outcome

The median pre-hospital pH was 7.19 (IQR 7.04−7.27), whilst the median admission pH was slightly higher at 7.24 (IQR 7.12–7.32). Patients with good neurological outcome had higher initial ROSC pH 7.26 (IQR 7.18–7.31) vs. 7.06 (IQR 6.93–7.19; *P* < 0.001) (see Supplementary material online, *Figure S3*). In total, 57/59 (97%) of patients with a ROSC pH <7.0 had poor neurological outcome. Multi-variable regression analysis incorporating other MIRACLE_2_ components showed that ROSC pH as a continuous variable remained an independent predictor of poor neurological outcome with an odds ratio of −5.07 [95% CI (−8.65, −1.85); *P* < 0.01] (*Table 4*). In this study, the optimal threshold for association with the primary endpoint to optimize both sensitivity and specificity was 7.22 for ROSC pH and so the conventional threshold of 7.20 was retained in the ROSC-MIRACLE_2_ score (see Supplementary material online, *Figure S4*).

### Performance of risk prediction models

*Table 2* and Supplementary material online, *Figure S2* highlight AUC, overall performance and feasibility across all three risk scores with multi-variable regression predictors of outcome and frequencies shown in Supplementary material online, *Tables S3and S4*. Feasibility of ROSC-MIRACLE_2_ calculation was 63.9%, the AUC was 0.89 (95% CI 0.85–0.92), with a Brier score of 0.137 and *R*^2^ of 0.550, reflecting strong model calibration and discrimination (*Table 2*). Intercepts and risk score coefficients were significant, indicating robust calibration and reliable differentiation of patient risk.

**Table 2:** AUC, *R*^2^, and brier scores for risk models

Metric	Pre-MIRACLE_2_	ROSC-MIRACLE_2_	MIRACLE_2_
AUC	0.88 [0.84, 0.92]	0.89 [0.85, 0.92]	0.89 [0.85, 0.92]
*R* ^2^	0.54 [0.44, 0.65]	0.57 [0.47, 0.67]	0.57 [0.46, 0.67]
Brier	0.14 [0.11, 0.16]	0.13 [0.11, 0.16]	0.13 [0.11, 0.16]

AUC, area under the receiver operating characteristic curve; *R*^2^, coefficient of determination; Brier, Brier calibration score.

The pre-MIRACLE_2_ score had a feasibility of calculation of 99.7% with an AUC of 0.88 (95% CI 0.84–0.92), a Brier score of 0.139, and an *R*^2^ of 0.535. Again, intercepts and risk score coefficients were significant, consistent with robust calibration and differentiation of patient risk (*Table 2*). For both ROSC-MIRACLE_2_ and pre-MIRACLE_2_, there was a stepwise increase in the primary endpoint of poor neurological outcome as the scores increased (see Supplementary material online, *Figure S2*).

The admission MIRACLE_2_ score demonstrated an AUC of 0.89 (95% CI 0.85–0.92), a Brier score of 0.134, and an *R*^2^ of 0.563. Model intercepts and risk score coefficients were again significant, confirming robust calibration and reliable discrimination between patient risk groups (*Table 2*).

In multi-variable analyses, incorporation of the pH enhanced predictive performance (see Supplementary material online, *Table S3*). In ROSC-MIRACLE_2_, a low post-ROSC pH showed a trend towards increased risk (β = 0.67, 95% CI −0.09, 1.43, *P* < 0.1), whilst in MIRACLE_2_, low pH on hospital arrival was a statistically significant predictor (β = 0.77, 95% CI 0.04, 1.50, *P* < 0.05).

### Discrimination performance of ROSC-MIRACLE2 and pre-MIRACLE2 scores by risk groups

For ROSC-MIRACLE_2_, four risk categories were defined as: low (score 0–2), intermediate (3–4), and high (5–6) and very high (≥7). Of 286 evaluable patients, 119 (41.6%) were classified as low risk, 65 (22.7%) as intermediate risk, 102 (35.7%) as high risk, and 34 (12.2%) as very high risk. Poor neurological outcome occurred in 13/119 (10.9%) low-risk patients, 33/65 (50.8%) intermediate-risk patients, and 90/102 (88.2%) high-risk patients. Patients classified as low risk (ROSC-MIRACLE_2_ ≤ 2) had a negative predictive value of 0.89 (95% CI 0.82–0.94), whilst those classified as high risk (ROSC-MIRACLE_2_ ≥ 5) had a positive predictive value of 0.88 (95% CI 0.82–0.94) (*Table 3*).

**Table 3:** Sensitivity, specificity, PPV, and NPV by risk score

Metric	>2	>4	>6
Pre-MIRACLE_2_			
*n* ≥ threshold (%)	155 (55.6)	66 (23.7)	12 (4.3)
Sensitivity	0.88 (0.82, 0.94)	0.44 (0.36, 0.52)	0.09 (0.04, 0.14)
Specificity	0.75 (0.68, 0.82)	0.95 (0.91, 0.99)	1.00 (1.00, 1.00)
PPV	0.77 (0.70, 0.83)	0.90 (0.81, 0.96)	1.00 (1.00, 1.00)
NPV	0.88 (0.81, 0.93)	0.65 (0.59, 0.71)	0.54 (0.48, 0.61)
ROSC-MIRACLE_2_			
*n* ≥ threshold (%)	167 (59.9)	100 (35.8)	34 (12.2)
Sensitivity	0.90 (0.85, 0.95)	0.66 (0.58, 0.74)	0.25 (0.18, 0.32)
Specificity	0.69 (0.62, 0.76)	0.92 (0.87, 0.96)	0.99 (0.98, 1.00)
PPV	0.73 (0.66, 0.80)	0.88 (0.82, 0.94)	0.97 (0.91, 1.00)
NPV	0.89 (0.82, 0.94)	0.75 (0.68, 0.81)	0.59 (0.53, 0.65)
MIRACLE_2_			
*n* ≥ threshold (%)	161 (57.7)	89 (31.9)	28 (10.0)
Sensitivity	0.89 (0.83, 0.94)	0.59 (0.51, 0.67)	0.21 (0.14, 0.28)
Specificity	0.72 (0.64, 0.79)	0.93 (0.89, 0.97)	1.00 (1.00, 1.00)
PPV	0.75 (0.68, 0.81)	0.89 (0.82, 0.95)	1.00 (1.00, 1.00)
NPV	0.88 (0.81, 0.93)	0.71 (0.65, 0.77)	0.58 (0.52, 0.64)

Values in brackets represent 95% confidence intervals. PPV, positive predictive value; NPV, negative predictive value.

For the pre-MIRACLE_2_ score, 131 patients (45.8%) were low risk (score 0–2), 89 (31.1%) intermediate risk (3–4), and 66 (23.1%) high risk (≥5). Poor neurological outcome occurred in 16/131 (12.2%) low-risk patients, 61/89 (68.5%) intermediate-risk patients, and 59/66 (89.4%) high-risk patients. The negative predictive value for low-risk patients was 0.88 (95% CI 0.81–0.93), whilst the positive predictive value for high-risk patients was 0.90 (95% CI 0.81–0.96) (*Table 3*), and the outcome at 1 month was −5.07 (95% CI −8.65, −1.85; *P* < 0.01) (*Table 4*).

**Table 4:** Logistic regression model with ROSC pH as predictor

	Unadjusted	Age and gender	MIRACLE2 covariates
Intercept	73.33***	78.94***	35.01**
	[55.68, 93.35]	[59.81, 100.71]	[11.62, 60.89]
ROSC pH	−10.25***	−10.95***	−5.07**
	[−13.03, −7.79]	[−13.98, −8.29]	[−8.65, −1.85]
Gender		−1.10**	−1.13*
		[−1.93, −0.30]	[−2.08, −0.21]
Age category 60–79		0.55+	0.45
		[−0.08, 1.20]	[−0.28, 1.19]
Age category 80+		1.49*	1.71*
		[0.30, 2.77]	[0.15, 3.32]
Non-shockable rhythm			0.81
			[−0.22, 1.90]
Not witnessed			−0.04
			[−1.02, 0.96]
Changing rhythm			0.93**
			[0.23, 1.64]
Adrenaline			1.75***
			[0.98, 2.55]
Non-reactive pupils			0.82+
			[−0.02, 1.67]
Num. Obs.	279	279	279

+ *P* < 0.1, * *P* < 0.05, ** *P* < 0.01, *** *P* < 0.001.

### Analysis of key sub-groups

The ROSC-MIRACLE_2_ risk score maintained a significant association with neurological outcome when stratified by STEMI [β = 0.75 (95% CI 0.50, 1.03)] and NSTEMI [β = 0.98 (95% CI 0.72, 1.30)]. There was a stronger relationship in the NSTEMI group [AUC 0.92 (0.88, 0.96), *R*^2^ = 0.66] compared with STEMI [AUC 0.83 (0.76, 0.89), *R*^2^ 0.42] (see Supplementary material online, *Table S5* and *Figures S5* and *S6*). ROSC-MIRACLE_2_ was also predictive of neurological outcome regardless of stratification to SCAI A at risk [β = 1.30 (0.66, 2.18)], vs. SCAI B-E CS [β = 0.80 (0.67, 1.13)]. ROSC-MIRACLE_2_ had equal performance in SCAI A [AUC 0.90 (95% CI 0.75, 0.98), *R*^2^ = 0.53] and SCAI B-E patient groups [AUC 0.87, (0.82, 0.91), *R*^2^ 0.51] (see Supplementary material online, tables*S6* and *S7* and *Figures S5* and *S7*).

## Discussion

In this prospective multi-centre study conducted across London, we show that pre-hospital stratification of resuscitated OHCA incorporating rapid point-of-care pH into the ROSC-MIRACLE_2_ score can accurately stratify this condition but with modest feasibility. A simplified modification of the ROSC-MIRACLE_2_ score without requirement for the pH component of the score, the pre-MIRACLE_2_ score, has equivalent performance and may enable higher feasibility and applicability in real-world practice.

Current international and multi-society endorsed guidelines continue to recommend conveyance of all non-traumatic OHCA patients direct to specialist CACs.^4,5,7^ However, the recent ARREST trial showed that expedited conveyance of all OHCA patients without STEMI direct to CACs compared with local EDs was not beneficial.^9^ The ARREST trial population was heterogeneous, which may support the development of tools to risk stratify this condition more effectively. The British Cardiovascular Intervention Society consensus statement instead recommended that OHCA patients with either STEMI or an initial shockable rhythm should be conveyed direct to CACs with preliminary observational data in consecutive patients suggesting a benefit.^11^ In view of lack of robust data, a recent meta-analysis provided a weak recommendation for adults with OHCA being cared for at CACs based on very low certainty of evidence.^26^ These recommendations may reflect a lack of clarity on the differential effect of expedited conveyance and provision of specialist cardiovascular care when stratified by initial rhythm and also inherent underlying neurological risk, which occurs in nearly two-thirds of OHCA patients. Pre-hospital stratification of poor neurological outcome may amplify advanced early critical care, inform expedited conveyance pathways, and more efficient provision of specialist CAC resources.

Several risk stratification tools have been reported for patients with OHCA; however, most have been developed and validated for use after hospital arrival or critical care admission, with limited evidence supporting their feasibility or performance in the pre-hospital setting.^12,13,27–30^ Tools derived from pre-hospital data, including those from the OHCAO registry, UB-ROSC and RACA, rely on complex multi-variable inputs and primarily predict intermediate endpoints such as ROSC or survival to hospital admission, limiting their applicability in routine, time-critical pre-hospital care.^31–33^ Similarly, machine-learning approaches with numerous inputs, remain difficult to operationalize at the point-of-care and lack external validation.^34^ In contrast, the ROSC-MIRACLE_2_ and pre-MIRACLE_2_ scores are practical, are validated in this study, and suitable for pre-hospital use, focusing on clinically meaningful neurological outcomes in OHCA patients.

In our study, we confirm that the ROSC-MIRACLE_2_ score with incorporation of a point-of-care pH measurement at the threshold of 7.20 has excellent performance for prediction of poor neurological outcome with an AUC of 0.89 and excellent calibration. Nearly half of patients had a ROSC-MIRACLE_2_ score of <3 with a negative predictive value of 0.88—representing a low-risk group appropriate for direct conveyance to a CAC, whilst a ROSC-MIRACLE_2_ score of >4 had a positive predictive value of 0.90. Equally, the pre-MIRACLE_2_ score, without the measurement of pH had satisfactory performance with an AUC of 0.88 and acceptable calibration. Patients with pre-MIRACLE_2_ score of <3 had a negative predictive value of 0.88, whilst a pre-MIRACLE_2_ of >5 had a positive predictive value of 0.90. However, the ROSC-MIRACLE2 score had modest feasibility, which confines its application in real-world practice limited by inability to gain vascular access, device error or failure to analyse the blood sample. The pre-MIRACLE_2_ score had substantially higher feasibility of calculation compared with ROSC-MIRACLE_2_ score in this study, primarily driven by this lack of requirement for point-of-care pH measurement. Both scores had similar performance compared with the MIRACLE_2_ score calculated on hospital admission.

The role of pre-hospital point-of-care measurement of pH and other parameters in the OHCA patient with ROSC has not been systematically evaluated but may be of clinical use, at least in selected cases. Several relatively small observational studies have shown POC testing is feasible during CPR in a pre-hospital setting and may be associated with earlier treatment of acid–base and electrolyte imbalances and subsequent ROSC.^35,36^ For the first time, whilst acknowledging constraints from feasibility, this study has incorporated POC data obtained at the time of ROSC into risk stratification tools with the potential for advanced pre-hospital decision making.

The pathophysiological importance of acid–base autoregulation in systems and cellular biology is well established.^37–40^ The prognostic value for pH levels after OHCA immediately after ROSC is unknown and current data supporting its association with outcome arise primarily from hospital admission studies, where this is obtained after a delay from the index event.^12,28,41,42^ In this study, we have shown that pH obtained at ROSC is independently associated with neurological outcome after OHCA. Furthermore, patients with a ROSC pH <7.0 had a very poor prognosis with poor neurological outcome occurring in 97% of patients. This presents a potential secondary threshold that may discriminate patients unlikely to have a good neurological outcome with a high degree of specificity. The classical optimal threshold of 7.20 on hospital admission reported in the original MIRACLE_2_ derivation study remained valid in at the time of ROSC in the RAPID-MIRACLE study. We may speculate that the acid–base balance of post-cardiac arrest syndrome is most optimally autoregulated above a pH threshold >7.20 and that any deterioration below this at any point after ROSC might lead to a cascade of dysregulatory mechanisms that are associated with poor outcome.

This study has several important implications for clinical practice, pathways of care and future research initiatives. Whilst previously derived and validated, this study now validates the pre-MIRACLE_2_ score in a prospective cohort of pre-hospital ROSC patients. The ROSC-MIRACLE_2_ score has potential value but is likely to be more suitable for systems of care with clearly established protocols for rapid point-of-care testing. Pre-hospital stratification, particularly with the pre-MIRACLE_2_ score, may now identify patients with requirement for more intensive pre-conveyance protocols or for studying of novel immediate post-ROSC therapeutic approaches. Importantly, pre-hospital stratification might identify sub-groups for expedited conveyance of patients to CACs with most reversibility and to prevent costly and potentially futile transfers of patients at a very poor prognosis. For example, patients with an initial shockable rhythm and those with an arrival MIRACLE_2_ score of ≤5 achieved most benefit from expedited transfer to specialist CACs in one small pilot study.^10^ Incorporation of the pre-MIRACLE_2_ score on scene, particularly for those with STEMI, may now be used for selecting patients for direct transfer to CACs. Finally, it is known that in resuscitated OHCA that downtime or its components have a relatively weaker discrimination of outcome compared with established and validated risk tools such as the MIRACLE_2_ score.^12,14^ Incorporation of the pre-MIRACLE_2_ score into protocols of care has the benefit of real-time calculation (with minimization of error) but to also improve the handover of patients as part of multi-disciplinary process. Pre-hospital stratification using these risk tools linked with targeted interventions now requires validation in randomized controlled trials.

### Limitations

This study has several limitations which require consideration. Whilst conducted as a prospective multi-centre study across a mature and large metropolitan network, the findings may not be generalizable to regions with different healthcare systems and settings. Owing to complexities of point-of-care measurement, this was protocolized to be performed by APP-CCs only, who, whilst attending a wide spectrum of OHCA cases in our region, selected the most appropriate cases based on clinical discretion and so recruited patients may therefore not be representative of the overall OHCA population. We purposefully only included suspected cardiac aetiology OHCA for our study, since the rationale for conveyance to specialist CACs is strongest for this group. Hence, the rates of shockable rhythm, STEMI, AED defibrillation, and good outcome are higher than in other studies, and the findings may not be generalizable to other aetiologies of this condition. These factors, combined with an inability to achieve a point-of-care measurement in over a third of cases, may have led to a selection bias. The methodology of the study cannot confirm exact feasibility of calculation of the pre-MIRACLE_2_ score compared with the ROSC-MIRACLE_2_ score but provides an estimation of this based on the data available from the pre-hospital and centre settings. Nevertheless, the findings of this study are of substantial importance to this field and may inform future studies of pre-hospital care for this condition.

## Conclusion

In the present study, pre-hospital application of the ROSC-MIRACLE_2_ score, with incorporation of point-of-care pH or a modified pre-MIRACLE_2_ score without pH enables early neurological risk stratification following resuscitated OHCA. Whilst incorporation of point-of-care pH improves precision, the simplified pre-MIRACLE_2_ score offers greater feasibility with comparable performance. Whether incorporation of pre-hospital risk stratification into pathways of care for resuscitated OHCA may improve patient selection, resource utilization, and outcomes warrants further study.

## Supplementary Material

zuag036_Supplementary_Data

## Data Availability

Because of the sensitive nature of the data collected for this study, requests to access the dataset from qualified researchers trained in human subject confidentiality protocols may be sent to the corresponding author at King’s College Hospital.
