# Supplementary material for: Point-of-care testing for pre-hospital stratification after out-of-hospital cardiac arrest: the RAPID-MIRACLE study
Source: Eur Heart J Acute Cardiovasc Care. 2026 Mar 30;15(3):220–8. doi: 10.1093/ehjacc/zuag036 (PMC13093581; doi:10.1093/ehjacc/zuag036)
Supplement: zuag036_Supplementary_Data [file zuag036_supplementary_data.docx]

**Point of Care testing for Pre-hospital stratification for Out of Hospital Cardiac Arrest – the RAPID-MIRACLE Study.**

**System Description and Standard EMS OHCA Response**

London Ambulance Service NHS Trust (LAS) is the primary provider of prehospital emergency care within Greater London, UK, covering a geographical area of approximately 607 square miles, with a resident population of 8.95 million people. LAS attends approximately 12,500 OHCA incidents annually, of which approximately 4,500 receive a resuscitation attempt.

Emergency calls received via the national emergency number (999) are triaged using the Medical Priority Dispatch System. Where cardiac arrest is identified, dispatcher-assisted CPR instructions are provided, with concurrent dispatch of ambulance resources. Cardiac arrest incidents receive a standardised response involving a minimum of four clinicians, at least one of whom is a paramedic trained to deliver Advanced Life Support (ALS) in line with national guidelines. A smartphone-based volunteer responder programme supports the response to OHCA in London.

Resuscitation is delivered on scene, unless there is a clear reversible cause necessitating hospital conveyance, and paramedics are permitted to withhold resuscitation where there are obvious signs of death, or conditions which would make resuscitation obviously futile (e.g. decapitation). Protocolised termination of resuscitation is permitted where there is no response to ALS interventions.

Additional clinical resources may be dispatched to the scene on a case-by-case basis including clinical managers (with mechanical CPR capability and additional clinical leadership training), and Advanced Paramedic Practitioners in Critical Care (APP-CC). Up to five APP-CC vehicles operate in London at any one time and these clinicians have received additional postgraduate training and operate with an extended scope of practice. They are selectively dispatched to patients who may benefit from enhanced critical care support, including OHCA patients, following interrogation of the call by an APP-CC in the ambulance control room, or request from EMS clinicians on scene.

| **Supplementary Table 1. Participating hospitals/NHS trusts in the RAPID-MIRACLE study** | | | | | | |
| --- | --- | --- | --- | --- | --- | --- |
| **Trust (Hospital sites)** | **Population catchment (million)** | **Number recruited by trust** | **London region** | **Access to 24/7 PCI  (site level)** | **On-site cardiac surgery  (site level)** | **Function  (site level)** |
| **King’s College Hospital NHS Foundation Trust** | 1.1 | 27 | South East | – | – | – |
| King’s College Hospital |  |  |  | Yes | Yes | Tertiary HAC |
| Princess Royal University Hospital |  |  |  | No | No | Secondary ED |
| **Lewisham and Greenwich NHS Trust** | 0.7 | 3 | South East | – | – | – |
| University Hospital Lewisham |  |  |  | No | No | Secondary ED |
| Queen Elizabeth Hospital |  |  |  | No | No | Secondary ED |
| **Chelsea and Westminster Hospital NHS Foundation Trust** | 0.6 | 2 | West | – | – | – |
| Chelsea and Westminster Hospital |  |  |  | No | No | Secondary ED |
| West Middlesex University Hospital |  |  |  | No | No | Secondary ED |
| **Guy’s and St Thomas’ NHS Foundation Trust** | 1.3 | 25 | Central | – | – | – |
| St Thomas’ Hospital |  |  |  | Yes | Yes | Tertiary HAC |
| **Kingston and Richmond NHS Foundation Trust** | 0.4 | 1 | South West | – | – | – |
| Kingston Hospital |  |  |  | No | No | Secondary ED |
| **St George’s University Hospitals NHS Foundation Trust** | 1.3 | 35 | South West | – | – | – |
| St George’s Hospital |  |  |  | Yes | Yes | Tertiary HAC |
| **Croydon Health Services NHS Trust** | 1.5 | 3 | South | – | – | – |
| Croydon University Hospital |  |  |  | No | No | Secondary ED |
| **London North West University Healthcare NHS Trust** | 0.4 | 16 | North West | – | – | – |
| Ealing Hospital |  |  |  | No | No | Secondary ED |
| Northwick Park Hospital |  |  |  | No | No | Tertiary HAC |
| **North Middlesex University Hospitals NHS Trust** | 0.4 | 2 | North | – | – | – |
| North Middlesex Hospital |  |  |  | No | No | Secondary ED |
| **Whittington Health NHS Trust** | 0.5 | 2 | North | – | – | – |
| The Whittington Hospital |  |  |  | No | No | Secondary ED |
| **Barts Health NHS Trust** | 2.6 | 58 | North East | – | – | – |
| St Bartholomew’s Hospital |  |  |  | Yes | Yes | Tertiary HAC |
| Royal London Hospital |  |  |  | No | No | Secondary ED |
| Newham University Hospital |  |  |  | No | No | Secondary ED |
| Whipps Cross Hospital |  |  |  | No | No | Secondary ED |
| **Barking, Havering and Redbridge University Hospitals NHS Trust** | 0.8 | 3 | East | – | – | – |
| King George Hospital |  |  |  | No | No | Secondary ED |
| Queen’s Hospital |  |  |  | No | No | Secondary ED |
| **Homerton Healthcare NHS Foundation Trust** | 0.3 | 2 | East | – | – | – |
| Homerton Hospital |  |  |  | No | No | Secondary ED |
| **The Hillingdon Hospitals NHS Foundation Trust** | 0.4 | 6 | West | – | – | – |
| Hillingdon Hospital |  |  |  | No | No | Secondary ED |
| **Imperial College Healthcare NHS Trust** | 2.0 | 51 | West | – | – | – |
| Hammersmith Hospital |  |  |  | Yes | Yes | Tertiary HAC |
| St Mary’s Hospital |  |  |  | No | No | Secondary ED |
| Charing Cross Hospital |  |  |  | No | No | Secondary ED |
| **Royal Free London NHS Foundation Trust** | 1.6 | 32 | North West | – | – | – |
| Royal Free Hospital |  |  |  | Yes | No | Tertiary HAC |
| Barnet Hospital |  |  |  | No | No | Secondary ED |
| **University College London Hospitals NHS Foundation Trust** | 1.0 | 3 | Central | – | – | – |
| University College Hospital |  |  |  | No | No | Secondary ED |
| **Royal Brompton and Harefield Hospitals NHS Trust** | 0.8 | 21 | North West | – | – | – |
| Harefield Hospital |  |  |  | Yes | Yes | Tertiary HAC |
| ED, Emergency Department; HAC, Heart Attack Centre; PCI, percutaneous coronary intervention. Recruitment numbers are reported at NHS trust level. | | | | | | |

**Collaborators**

The RAPID-MIRACLE study was conducted as a multicentre collaboration involving the following principal investigators and NHS trusts: Barking, Havering and Redbridge University Hospitals NHS Trust, Dr Darryl Wood; Barts Health NHS Trust, Dr Paul Rees; Chelsea and Westminster Hospital NHS Foundation Trust, Dr Sukhjinder Nijjer; Croydon Health Services NHS Trust, Dr Kostantinos Zacharias; Epsom and St Helier University Hospitals NHS Trust, Dr Nikesh Malik; Guy’s and St Thomas’ NHS Foundation Trust, Dr Tiffany Patterson; Homerton Healthcare NHS Foundation Trust, Dr Victor Voon; The Hillingdon Hospitals NHS Foundation Trust, Dr Gareth Rosser; Imperial College Healthcare NHS Trust, Dr Iqbal Malik; King’s College Hospital NHS Foundation Trust, Dr Nilesh Pareek; Kingston and Richmond NHS Foundation Trust, Dr Rupert Williams; Lewisham and Greenwich NHS Trust, Dr Antonis Pavlidis; London North West University Healthcare NHS Trust, Dr Aamir Ali; North Middlesex University Hospitals NHS Trust, Rachel Vincent; Royal Brompton and Harefield Hospitals NHS Trust, Dr Miles Dalby; Royal Free London NHS Foundation Trust, Dr Sundeep Kalra; St George’s University Hospitals NHS Foundation Trust, Dr Sam Firoozi; University College London Hospitals NHS Foundation Trust, Dr Robert Bell; and Whittington Health NHS Trust, Dr David Brull.

This list is ordered alphabetically by NHS trust. The authors also acknowledge the substantial contribution of the London Ambulance Service, including Advanced Paramedic Practitioners in Critical Care (APP-CC) teams, and additional clinicians and research staff at participating centres who supported patient identification, enrolment, data collection, and study delivery but are not individually named; we are extremely grateful for their efforts.

| **Supplementary Table 2. STROBE Statement—checklist of items that should be included in reports of observational studies (1)** | | | |
| --- | --- | --- | --- |
|  | Item No. | Recommendation | Page No. |
| Title and abstract | 1 | (*a*) Indicate the study’s design with a commonly used term in the title or the abstract | 1 |
|  |  | (*b*) Provide in the abstract an informative and balanced summary of what was done and what was found | 2 |
| Introduction | | | |
| Background/rationale | 2 | Explain the scientific background and rationale for the investigation being reported | 4 |
| Objectives | 3 | State specific objectives, including any prespecified hypotheses | 4 |
| Methods | | | |
| Study design | 4 | Present key elements of study design early in the paper | 5 |
| Setting | 5 | Describe the setting, locations, and relevant dates, including periods of recruitment, exposure, follow-up, and data collection | 5 |
| Participants | 6 | (*a*) *Cohort study*—Give the eligibility criteria, and the sources and methods of selection of participants. Describe methods of follow-up  *Case-control study*—Give the eligibility criteria, and the sources and methods of case ascertainment and control selection. Give the rationale for the choice of cases and controls  *Cross-sectional study*—Give the eligibility criteria, and the sources and methods of selection of participants | 5-6 |
|  |  | (*b*) *Cohort study*—For matched studies, give matching criteria and number of exposed and unexposed  *Case-control study*—For matched studies, give matching criteria and the number of controls per case |  |
| Variables | 7 | Clearly define all outcomes, exposures, predictors, potential confounders, and effect modifiers. Give diagnostic criteria, if applicable | 6-7 |
| Data sources/ measurement | 8* | For each variable of interest, give sources of data and details of methods of assessment (measurement). Describe comparability of assessment methods if there is more than one group | 6-7 |
| Bias | 9 | Describe any efforts to address potential sources of bias | 7 |
| Study size | 10 | Explain how the study size was arrived at | 7 |

|  | **Supplementary Table 3.**  **Multivariable regression analysis for predictors of Pre-MIRACLE_2_, ROSC-MIRACLE_2_ and MIRACLE_2_ scores** | | | |
| --- | --- | --- | --- | --- |
| **Predictors** | | **Pre-MIRACLE_2_** | **ROSC-MIRACLE_2_** | **MIRACLE_2_** |
| **Intercept** | | -0.13  (-1.63, 1.43) | -0.65  (-2.29, 1.02) | -0.57  (-2.16, 1.07) |
| **Age Category 60-79** | | 0.32  (-0.36, 1.01) | 0.39  (-0.30, 1.09) | 0.46  (-0.24, 1.18) |
| **Age Category 80+** | | 1.41 +  (-0.04, 2.93) | 1.52*  (0.05, 3.05) | 1.53*  (0.08, 3.06) |
| **Shockable Rhythm** | | -1.28 **  (-2.27, -0.36) | -1.11*  (-2.12, -0.17) | -1.24*  (-2.26, -0.30) |
| **Witnessed Arrest** | | -0.21  (-1.17, 0.71) | -0.21  (-1.17, 0.72) | -0.17  (-1.13, 0.76) |
| **Changing Rhythm** | | 1.01**  (0.33, 1.70) | 1.01**  (0.32, 1.70) | 0.98 **  (0.29, 1.68) |
| **Adrenaline During Arrest** | | 2.28***  (1.59, 3.01) | 2.04***  (1.29, 2.81) | 2.11***  (1.40, 2.86) |
| **Pupils Reactive** | | -1.08**  (-1.88, -0.31) | -0.91*  (-1.73, -0.11) | -0.91*  (-1.73, -0.11) |
| **ROSC low pH** | | - | 0.67 +  (-0.09, 1.43) | - |
| **Arrival low pH** | | - | - | 0.77*  (0.04, 1.50) |
| **No. of observations** | | 279 | 279 | 279 |
|  | Values are reported β coefficients with corresponding 95% confidence intervals; Low pH, pH <7.20; + p<0.1, * p<0.05, ** p<0.01, *** p<0.001 | | | |

| **Supplementary Table 4. Distribution of PRE-MIRACLE_2_, ROSC-MIRACLE_2_, and MIRACLE_2_ scores.** | | | |
| --- | --- | --- | --- |
| Score | PRE-MIRACLE_2_ | ROSC-MIRACLE_2_ | MIRACLE_2_ |
| 0 | 37 (13.3%) | 34 (12.2%) | 36 (12.9%) |
| 1 | 53 (19.0%) | 45 (16.1%) | 42 (15.1%) |
| 2 | 34 (12.2%) | 33 (11.8%) | 40 (14.3%) |
| 3 | 42 (15.1%) | 29 (10.4%) | 27 (9.7%) |
| 4 | 47 (16.8%) | 37 (13.3%) | 44 (15.8%) |
| 5 | 28 (10.0%) | 39 (14.0%) | 35 (12.5%) |
| 6 | 26 (9.3%) | 28 (10.0%) | 27 (9.7%) |
| 7 | 10 (3.6%) | 22 (7.9%) | 18 (6.5%) |
| 8 | 2 (0.7%) | 10 (3.6%) | 8 (2.9%) |
| 9 | 0 (0.0%) | 2 (0.7%) | 2 (0.7% |

| **Supplementary Table 5. AUC, R2 and Brier Score for Pre-MIRACLE_2_, ROSC-MIRACLE_2_ and MIRACLE_2_ scores by STEMI and Non-STEMI** | | | | | | |
| --- | --- | --- | --- | --- | --- | --- |
|  | **Pre-MIRACLE_2_** | **ROSC-MIRACLE_2_** | **MIRACLE _2_** | **ROSC - Pre** | **MIR_2_ - ROSC** | **MIR_2_ - Pre** |
| Measure: AUC | | | | | | |
| STEMI | 0.83 [0.75, 0.89] | 0.83 [0.76, 0.89] | 0.83 [0.77, 0.89] | 0.00 [-0.02, 0.03] | -0.00 [-0.02, 0.02] | 0.00 [-0.02, 0.02] |
| Non-STEMI | 0.91 [0.86, 0.95] | 0.92 [0.88, 0.96] | 0.93 [0.88, 0.96] | 0.01 [-0.00, 0.03] | 0.00 [-0.01, 0.01] | 0.01 [-0.00, 0.03] |
| Measure: R2 | | | | | | |
| STEMI | 0.40 [0.24, 0.57] | 0.42 [0.27, 0.58] | 0.42 [0.28, 0.58] | 0.02 [-0.04, 0.07] | -0.01 [-0.05, 0.04] | 0.01 [-0.03, 0.06] |
| Non-STEMI | 0.64 [0.49, 0.76] | 0.66 [0.53, 0.78] | 0.67 [0.53, 0.79] | 0.02 [-0.02, 0.06] | 0.01 [-0.03, 0.05] | 0.03 [-0.01, 0.08] |
| Measure: Brier | | | | | | |
| STEMI | 0.16 [0.12, 0.20] | 0.16 [0.12, 0.19] | 0.16 [0.12, 0.19] | -0.00 [-0.01, 0.01] | 0.00 [-0.01, 0.01] | 0.00 [-0.01, 0.01] |
| Non-STEMI | 0.11 [0.08, 0.15] | 0.11 [0.08, 0.14] | 0.10 [0.07, 0.14] | -0.00 [-0.01, 0.01] | -0.00 [-0.01, 0.00] | -0.01 [-0.02, 0.00] |

| **Supplementary Table 6. AUC, R2 and Brier Score for Pre-MIRACLE, ROSC-MIRACLE_2_ and MIRACLE_2_ scores by SCAI A and SCAI B-E.** | | | | | | |
| --- | --- | --- | --- | --- | --- | --- |
| **SCAI** | **Pre-MIRACLE** | **ROSC-MIRACLE_2_** | **MIRACLE_2_** | **ROSC-Pre** | **MIR_2_- ROSC** | **MIR_2_ - Pre** |
| AUC | | | | | | |
| SCAI A | 0.88 [0.73, 0.97] | 0.90 [0.75, 0.98] | 0.87 [0.71, 0.97] | 0.02 [-0.02, 0.06] | -0.03 [-0.08, 0.01] | -0.01 [-0.03, 0.00] |
| SCAI B-E | 0.86 [0.81, 0.91] | 0.87 [0.82, 0.91] | 0.87 [0.82, 0.92] | 0.01 [-0.01, 0.02] | 0.00 [-0.01, 0.02] | 0.01 [-0.00, 0.02] |
| R^2^ | | | | | | |
| SCAI A | 0.48 [0.18, 0.79] | 0.53 [0.20, 0.80] | 0.46 [0.16, 0.78] | 0.04 [-0.08, 0.16] | -0.06 [-0.17, 0.05] | -0.02 [-0.06, -0.00] |
| SCAI B-E | 0.50 [0.37, 0.63] | 0.51 [0.40, 0.64] | 0.52 [0.40, 0.65] | 0.02 [-0.02, 0.05] | 0.01 [-0.02, 0.04] | 0.02 [-0.01, 0.06] |
| Brier Score | | | | | | |
| SCAI A | 0.09 [0.04, 0.14] | 0.09 [0.04, 0.13] | 0.09 [0.04, 0.15] | -0.00 [-0.02, 0.02] | 0.01 [-0.01, 0.03] | 0.00 [0.00, 0.01] |
| SCAI B-E | 0.14 [0.11, 0.17] | 0.14 [0.11, 0.17] | 0.14 [0.11, 0.17] | 0.00 [-0.01, 0.01] | -0.00 [-0.01, 0.00] | -0.00 [-0.01, 0.00] |

| **Supplementary Table 7. Sensitivity, Specificity, PPV and NPV by MIRACLE score thresholds.** | | | | |
| --- | --- | --- | --- | --- |
| **SCAI** | **Metric** | **Threshold 3** | **Threshold 5** | **Threshold 7** |
| Score: Pre-MIRACLE_2_ | | | | |
| SCAI 1 | N ≥ threshold (%) | 15 (25.4) | 2 (3.4) | 0 (0.0) |
|  | Sensitivity | 0.75 (0.41, 1.00) | 0.17 (0.00, 0.44) | 0.00 (0.00, 0.00) |
|  | Specificity | 0.85 (0.74, 0.94) | 1.00 (1.00, 1.00) | 1.00 (1.00, 1.00) |
|  | PPV | 0.53 (0.26, 0.80) | 1.00 (1.00, 1.00) | NA ( NA, NA) |
|  | NPV | 0.93 (0.84, 1.00) | 0.84 (0.74, 0.93) | 0.81 (0.71, 0.91) |
| SCAI 2-5 | N ≥ threshold (%) | 140 (63.6) | 63.5 (28.9) | 12 (5.5) |
|  | Sensitivity | 0.90 (0.84, 0.94) | 0.46 (0.38, 0.54) | 0.09 (0.05, 0.14) |
|  | Specificity | 0.70 (0.60, 0.79) | 0.93 (0.87, 0.97) | 1.00 (1.00, 1.00) |
|  | PPV | 0.79 (0.72, 0.86) | 0.89 (0.81, 0.96) | 1.00 (1.00, 1.00) |
|  | NPV | 0.84 (0.76, 0.91) | 0.57 (0.50, 0.64) | 0.46 (0.39, 0.53) |
| Score: ROSC-MIRACLE_2_ | | | | |
| SCAI 1 | N ≥ threshold (%) | 19 (32.2) | 4 (6.8) | 0 (0.0) |
|  | Sensitivity | 0.92 (0.70, 1.00) | 0.36 (0.09, 0.73) | 0.00 (0.00, 0.00) |
|  | Specificity | 0.81 (0.69, 0.92) | 1.00 (1.00, 1.00) | 1.00 (1.00, 1.00) |
|  | PPV | 0.53 (0.28, 0.75) | 1.00 (1.00, 1.00) | NA ( NA, NA) |
|  | NPV | 0.98 (0.92, 1.00) | 0.87 (0.77, 0.96) | 0.81 (0.71, 0.91) |
| SCAI 2-5 | N ≥ threshold (%) | 148 (67.3) | 96 (43.6) | 33.5 (15.2) |
|  | Sensitivity | 0.90 (0.85, 0.95) | 0.68 (0.59, 0.76) | 0.26 (0.20, 0.35) |
|  | Specificity | 0.62 (0.53, 0.73) | 0.88 (0.81, 0.94) | 0.99 (0.97, 1.00) |
|  | PPV | 0.76 (0.69, 0.83) | 0.88 (0.81, 0.94) | 0.97 (0.90, 1.00) |
|  | NPV | 0.83 (0.75, 0.92) | 0.68 (0.59, 0.76) | 0.51 (0.44, 0.58) |
| Score: MIRACLE_2_ | | | | |
| SCAI 1 | N ≥ threshold (%) | 16 (27.1) | 2 (3.4) | 0 (0.0) |
|  | Sensitivity | 0.75 (0.41, 1.00) | 0.17 (0.00, 0.44) | 0.00 (0.00, 0.00) |
|  | Specificity | 0.83 (0.72, 0.94) | 1.00 (1.00, 1.00) | 1.00 (1.00, 1.00) |
|  | PPV | 0.50 (0.24, 0.77) | 1.00 (1.00, 1.00) | NA ( NA, NA) |
|  | NPV | 0.93 (0.84, 1.00) | 0.84 (0.74, 0.93) | 0.81 (0.71, 0.91) |
| SCAI 2-5 | N ≥ threshold (%) | 145 (65.9) | 87 (39.5) | 27 (12.3) |
|  | Sensitivity | 0.90 (0.85, 0.95) | 0.62 (0.54, 0.70) | 0.22 (0.16, 0.29) |
|  | Specificity | 0.66 (0.56, 0.76) | 0.90 (0.84, 0.95) | 1.00 (1.00, 1.00) |
|  | PPV | 0.77 (0.70, 0.84) | 0.89 (0.82, 0.94) | 1.00 (1.00, 1.00) |
|  | NPV | 0.84 (0.76, 0.92) | 0.65 (0.57, 0.73) | 0.50 (0.43, 0.56) |

**REFERENCES**

1. STROBE. STROBE Statement—checklist of items that should be included in reports of observational studies 2024 [Available from: <https://www.strobe-statement.org>.
